# Preparation and Characterization of Ni-Doped TiO_2_ Materials for Photocurrent and Photocatalytic Applications

**DOI:** 10.1100/2012/127326

**Published:** 2012-04-24

**Authors:** Ibram Ganesh, A. K. Gupta, P. P. Kumar, P. S. C. Sekhar, K. Radha, G. Padmanabham, G. Sundararajan

**Affiliations:** Laboratory for Photoelectrochemical (PEC) Cells and Advanced Ceramics, International Advanced Research Centre for Powder Metallurgy and New Materials (ARCI), Balapur PO, Hyderabad 500005, India

## Abstract

Different amounts of Ni-doped TiO_2_ (Ni = 0.1 to 10%) powders and thin films were prepared by following a conventional coprecipitation and sol-gel dip coating techniques, respectively, at 400 to 800°C, and were thoroughly characterized by means of XRD, FT-IR, FT-Raman, DRS, UV-visible, BET surface area, zeta potential, flat band potential, and photocurrent measurement techniques. Photocatalytic abilities of Ni-doped TiO_2_ powders were evaluated by means of methylene blue (MB) degradation reaction under simulated solar light. Characterization results suggest that as a dopant, Ni stabilizes TiO_2_ in the form of anatase phase, reduces its bandgap energy, and adjusts its flat band potentials such that this material can be employed for photoelectrochemical (PEC) oxidation of water reaction. The photocatalytic activity and photocurrent ability of TiO_2_ have been enhanced by doping of Ni in TiO_2_. The kinetic studies revealed that the MB degradation reaction follows the Langmuir-Hinshelwood first-order reaction relationship.

## 1. Introduction

Recently, the photoelectrochemical (PEC) conversion of CO_2_ into methanol and photoelectrolysis of water into H_2_ gas have received a great deal of attention from the scientific community [1–5]. These reactions are popularly called as artificial photosynthesis, which can transform solar light into chemical energy. These reactions require thermodynamic energy inputs of 1.23 and 1.5 eV, respectively [4–9]. Greater energy inputs are required to make up the losses due to band bending (necessary in order to separate charge at the semiconductor surface), resistance losses, and overvoltage potentials. The most frequently studied material for the photoelectrode is TiO_2_ [6, 7]. Given its indirect bandgap transition, the TiO_2_ in the form of anatase phase is the most preferred one among its three crystal modifications (i.e., anatase, rutile, and brookite) for PEC applications. Despite its high bandgap of 3 eV, TiO_2_ is the favored material owing to its high corrosion resistance. The maximum value obtained for the photovoltage of a PEC cell equipped with a TiO_2_ photoanode is ~0.7–0.9 eV [10]. This implies that the single-electrode TiO_2_-based PEC cell requires some amount of an external bias voltage for performing artificial photosynthesis reactions [1–10]. For the first time, Nozik [11, 12] successfully photooxidized water without employing any external bias voltage by utilizing simultaneously n-type TiO_2_ and p-GaP as photoanode and photocathode, respectively. As p-GaP undergoes photocorrosion; there has been a continuous quest for stable oxide-based p-type semiconducting materials for PEC applications [8]. Given its superior corrosion resistance and most promising flat band potentials suitable for artificial photosynthesis reactions, more focus was made on TiO_2_ to convert it from n-type conducting to p-type, and to reduce its bandgap energy and recombination of its photogenerated electron-hole pairs [13–20]. The p-type conducting behavior was occasionally observed for TiO_2_ after doping with certain metal ions such as Fe^3+^, Co^3+^, Ni^2+^, Cu^1+^ [8, 13–20]. The change of semiconducting behavior has been attributed to the heterounions formed between n-type TiO_2_ and p-type metal oxide dopant [18]. Among various transition metal ion dopants, Ni^2+^ appears to be a more efficient dopant for TiO_2_ as it has improved the photocatalytic activity of certain semiconductor photocatalysts [21, 22]. The reason for this enhancement has been tentatively attributed to the suppression of recombination of electron-hole pairs on the surface of the TiO_2_ catalyst by low valence Ni^2+^ ions [23]. In another study, Devi et al. [24] evaluated the photocatalytic activity of 0.08% Ni^2+^-doped TiO_2_ powders by degrading methyl orange (MO), an azo dye, under solar light and found considerable activity for this reaction. This improved activity has been attributed to the enhanced separation of photogenerated electron-hole pairs due to the presence of Ni^2+^ ions in TiO_2_. Although many published articles deal with Ni-doped TiO_2_ materials, they provide contradicting information as far as the effect of Ni-doping on photocurrent and photocatalytic behavior of TiO_2_ is concerned [16, 20, 23–31]. Furthermore, photocurrent and flat band potential properties of Ni-doped TiO_2_ materials are hardly studied. The photocatalytic degradation of methylene blue (MB) over Ni-doped TiO_2_ was not studied so far using light that is equivalent the light reaching the surface of the earth.

 In view of the above, different amounts of Ni-doped TiO_2_ powders and thin films were prepared, calcined in air and characterized by the following various techniques such as, XRD, SEM, EDAX, FT-IR, FT-Raman, DRS, UV-Vis, BET surface area, zeta potential, photocurrent, flat band potential, photocatalytic activity, and bandgap energy value measurements. The characterization results are presented and discussed in this paper to reveal the effects of Ni-doping on solar-light-induced photocurrent and photocatalytic behavior of TiO_2_.

## 2. Materials and Methods

### 2.1. Synthesis of Ni-Doped TiO_**2**_ Powders

Different amounts of Ni-doped TiO_2_ (Ni = 0, 0.1, 0.2, 0.3, 0.4, 0.5, 0.6, 0.7, 0.8, 0.9, 1.0, 2.0, 3.0, 4.0, 5.0, 6.0, 7.0, 8.0, 9.0, and 10.0 at. wt.%) powders were prepared by following a conventional coprecipitation technique using ammonia solution as an hydrolyzing agent [34, 35]. The concentration of Ni-doping in TiO_2_ is represented in terms of atomic weight percent. In a typical experiment, cold titanium tetrachloride (TiCl_4_, Fluka, AR grade) was first digested in cold concentrated HCl (AR Grade, Loba Chemie, Mumbai, India) and was subsequently diluted with deionized water. To this solution, the requisite amount of nickel nitrate (Ni(NO_3_)_2_·6H_2_O, AR Grade, Loba Chemie, Mumbai, India) dissolved separately in deionized water was added. The resultant solution was hydrolyzed by adding the 50% diluted ammonia solution drop by drop until the precipitate (at approximately about pH 9) is obtained. This precipitate was heated for about 12 h at 90°C to facilitate the aging process. The resultant precipitate was filtered off and washed thoroughly with doubly distilled water until no chloride ions could be detected with Ag^+^ ions in the filtrate. The resultant pure Ni(OH)_2_-Ti(OH)_4_ coprecipitate was oven dried at 120°C for 24 h and calcined at 550°C for 6 h in a electrical furnace in an open-air atmosphere. Some portions of the dried precipitates were also calcined at 400°C, 500°C, 600°C, 700°C, and 800°C for 6 h to study the thermal stability of these powders.

### 2.2. Preparation of Ni-Doped TiO_**2**_ Optically Transparent Thin Films

Optically transparent Ni-doped TiO_2_ (Ni = 0, 0.1, 0.5, 1, 5, and 10%) thin films were also prepared on conductive fluorine-doped tin-oxide- (FTO-) coated soda-lime glass (Glass Product, TEC 8, FIC Fine Chemicals, China having a resistivity of 6–9 ohm·sq) and nonconductive fused silica (FS) (Chhaperia International Company, Bangolore, India) slides of 75 × 25 × 1 mm size by following a conventional sol-gel dip coating technique [36]. Titanium (IV) iso-propoxide (TIP, Ti(OCH(CH_3_)_2_)_4_, AR grade, Alfa-Aeser) and nickel nitrate (Ni(NO_3_)_2_·6H_2_O) were used as sources of Ti and Ni, respectively. In a typical experiment, initially, a transparent colloidal sol was prepared by mixing TIP, ethanol, and di-ethanolamine in 1 : 5 : 0.5 volume ratio (for pure TiO_2_ thin films) [36]. Different types of transparent sols prepared were stirred for 4 h at room temperature to enhance the reactions between diethanolamine and TIP, and to increase the viscosity of the sols. Both conductive and nonconductive glass slides were cleaned for 15 min each with 0.1 M HNO_3_, 0.1 M NaOH and H_2_O in a row in an ultrasonic bath. The washed and dried (at 90°C in a vacuum of 2.5 × 10^−2^ torr) glass slides were dip coated (only one side) in the prepared transparent precursor sols by following a withdrawal speed of 1 mm/s. These dip-coated glass slides were dried under ambient conditions for 1 h prior to treating them in open-air atmosphere for 30 min at 500 and 600°C, respectively. In the case of conductive glass slides, the dip coating and calcination steps were repeated twice to enhance the film thickness.

### 2.3. Photocatalytic Activity Evaluation

The photocatalytic activity of Ni-doped TiO_2_ powders was evaluated by performing methylene blue (MB) (Loba-Chemie, Mumbai, India) degradation reactions under simulated solar light. In a typical experiment, 200 mL of 0.01, 0.02, or 0.03 mM aqueous MB solution and 0.24 g fine powder catalyst were taken in a glass dish (150 mm diameter × 75 mm height, Borosil, India) and exposed to simulated solar light (Osram 1000 W Xenon short arc display/optic lamp, XBO, Germany, installed in a light-condensing lamp housing, Model number: SS-1 K, Sciencetech Inc., Canada) for 0.5 to 4 h. The incident light was passed through an AM1.5D air-mass filter and the intensity of the incident light was measured to be about 44.42 mW/cm^2^ [37]. The reaction mixture solution was initially stirred for about 2 h in the dark to reach the adsorption equilibrium. During light irradiation, the reaction mixture was continuously stirred on a magnetic stirrer (Model: 5MLH, Remi, Mumbai, India) and aliquots of the reaction mixture were collected at regular time intervals. The leftover MB concentrations were estimated with the help of UV-vis spectrophotometer (Lambda 650, Perkin Elmer, Massachusetts, USA). The maximum absorption peak (*λ*_max⁡_) of MB at about 666 nm was considered to estimate the remaining concentration of MB in aqueous solutions [37].

### 2.4. Photocurrent and Flat Band Potential Measurements

The photoelectrochemical measurements of Ni-doped TiO_2_ (Ni = 0, 0.1, 0.5, 1, 5, and 10%) thin films were performed in a specially designed three-electrode reactor cell having a flat quartz window to facilitate the horizontal transmittance of light to the working electrode (1 cm^2^ surface area). The 1 M NaOH solution was used as the supporting electrolyte, Ag/AgCl (3 M NaCl) as a reference electrode, platinum gauze (1 cm × 1 cm) as a counter electrode, and a bipotentiostat (PARSTAT 2273, potentiostat/galvanostat/FRA, Oak Reidge, USA) for measuring the photocurrent [38]. The light source was a Xenon arc lamp of 500 W (Solar Simulator, Oriel 91160) having AM 1.5G filter and a monochromator (Newport-74125 model) with a bandwidth of 5 nm.

The electron energy states of quasi-Fermi levels (_*n*_ *E*_F_*) of Ni-doped TiO_2_ (Ni = 0.1, 0.5, 1, 5, and 10%) powders were measured using methyl viologen dichloride (MV^2+^, *E*_red_ = −0.445 V versus NHE) as a pH-independent redox system [39, 40]. The obtained pH_0_ values were converted to the Fermi potentials at pH 7 by the equation *E_*f*_ (pH 7) = −0.445 + 0.059 (pH_0_ −7). Reproducibility of the pH_0_ values was better than 0.1 pH units. In a typical experiment, 30 mg of catalyst powder and 6 mg of MV^2+^ were suspended in a specially designed glass reactor cell having a flat quartz window (~75 mL of 0.1 M KNO_3_). A platinum flag of 1 cm^2^ area and Ag/AgCl were served as working and reference electrodes, respectively, and a pH meter was used for recording the proton concentration. Prior to measurements, the solutions were purged with argon gas (>200 mL per min) for >2 h and continued the same during potential measurements. Initially, the pH of the solution was adjusted to pH 1-2 prior to recording readings using the solutions of HNO_3_ (0.1 M). The light source was the same as used in the PEC measurements. Stable photovoltages were recorded after about 30 min of changing the pH value using a multimeter (Agilent, Singapore) [39, 40]. All the potentials are presented against NHE reference in this paper.

### 2.5. Characterization

A Gemini Micromeritics analyzer (Micromeritics, Norcross, USA) was used for Brunauer-Emmett-Teller (BET) surface area measurements. The BET surface area was measured by nitrogen physisorption at liquid nitrogen temperature (–196°C) by considering 0.162 nm^2^ as the area of cross-section of N_2_ molecule. Prior to measurements, the samples were evacuated (up to 1 × 10^−3^ Torr) at 180°C for 2 h. Phase analysis, crystallite size, and lattice parameter values of the powders were determined by X-ray diffraction (Bruker's D8 advance system, Bruker's AXS, GmbH, Germany) using Cu K*α* radiation source. To obtain quantitative information of phases, the most intense peak of the individual phase was taken into consideration. The peak heights of all the phases were summed up and the percentage concentration of a particular phase was estimated from the ratio of the strongest peak of that phase to the sum of various phases present in a given system [41, 42]. The crystallite sizes of the powders were estimated with the help of Debye-Scherrer equation (〈*L*〉_*hkl*_ = *Kλ*/*β*_*hkl*_cos⁡ *θ*; where *K* is a constant taken as 1 and **β** is the integral breadth that depends on the width of the particular *hkl* plane; *λ* = 1.5406 Å, the wavelength of the Cu K*α* source; and *θ* is the Bragg's angle) using the XRD data of the strongest reflection of the major phase [41, 42]. The lattice parameters were determined using the equation 1/*d*_(*hkl*)_^2^ = (*h* ^2^ + *k* ^2^)/*a* ^2^ + *l* ^2^/*c* ^2^ (where the value of *d*_(*h*,*k*,*l*)_, for an XRD peak was determined from Bragg's law, 2*d*_(*hkl*)_ sin⁡ *θ* = *nλ*). Here, *hkl* is the crystal plane indices, *d*_(*hkl*)_ is the distance between crystal planes of (*hkl*), and *a*, *c* are the lattice parameters (for tetragonal anatase and rutile phases of TiO_2_: *a* = *b* ≠ *c*). The planes, (101) and (200) for anatase (ICDD File number: 03-065-5714), and the planes, (110) and (211) for rutile (ICDD File number: 03-065-1118), were considered while calculating the lattice parameters values. The content ratio of the anatase to rutile phase in titanium dioxide was roughly estimated using the following equation [43, 44]:

(1) $$\begin{array}{c} \text{content}\;\text{ratio}\;\text{of}\;\text{anatase}\;\left(\text{\%}\right)=\frac{100}{\left(1+1.265I_{A}/I_{R}\right)},\\ \text{content}\;\text{ratio}\;\text{of}\;\text{rutile}\;\left(\text{\%}\right)=\frac{1-100}{\left(1+1.265I_{A}/I_{R}\right)}. \end{array}$$

The percentage transmittance and absorbance of TiO_2_ thin films were measured using a UV-Vis spectrophotometer (Lambda 650, Perkin Elmer, Massachusetts, USA) in the wavelength range of 200–800 nm. The micrographs of the powders were examined using a scanning electron microscope (JSM-5410, JEOL, Japan) with an energy-dispersive scanning (Sigma 3.42 Quaser, Kevex, USA) attachment for qualitative and quantitative microanalysis. The FT-IR spectra were recorded on an FT-IR 1650 Perkin-Elmer Spectrometer (4000–200 cm^−1^) using KBr pellets. The Fourier-transform Raman spectra (FT-Raman) were recorded on a triple subtractive Jobin Yvon T64000 Raman spectrometer equipped with a liquid-nitrogen-cooled charge-coupled device (CCD) detector. Diffuse reflectance spectra (DRS) of the powders were recorded on a Shimadzu UVPC 3101 spectrophotometer equipped with an integration sphere. The powder samples were directly placed into a cylindrical sample port holder (3 cm diameter, 1 mm deep). The zeta-potentials of powders in 10^−3^ M KCl aqueous solutions were measured on a zeta meter (Zeta Meter Inc., USA). Dilute HNO_3_ and TMAH solutions were used for pH adjustment. The bandgap energies of Ni-doped TiO_2_ powders and thin films were calculated using the absorbance data obtained from DRS and UV-Vis spectroscopy techniques, respectively, by following the Tauc's relation [(*αhν*) = *C*(*hν*  −*E*_*g*_)^*n*^], where *C* is a constant, *α* is absorption coefficient (*α* = *A*/*L*, *L* is the thickness of the thin film, and *A* is the absorbance; *A* = *αL*, Lambert-Beers law), *E*_*g*_ is the average bandgap of the material and “*n*” depends on type of transition (2 for indirect bandgap and 1/2 for direct bandgap), *h* is the Plank's constant (6.626 × 10^−34^ J-s), and *v* is the frequency of photons [32, 33]. The direct and indirect average bandgap transition energies were estimated from the intercepts of linear portion of the (*αhν*)^2^ or (*αhν*)^1/2^ versus *hν* of plots, respectively.

## 3. Results and Discussion

**Figure 1:**
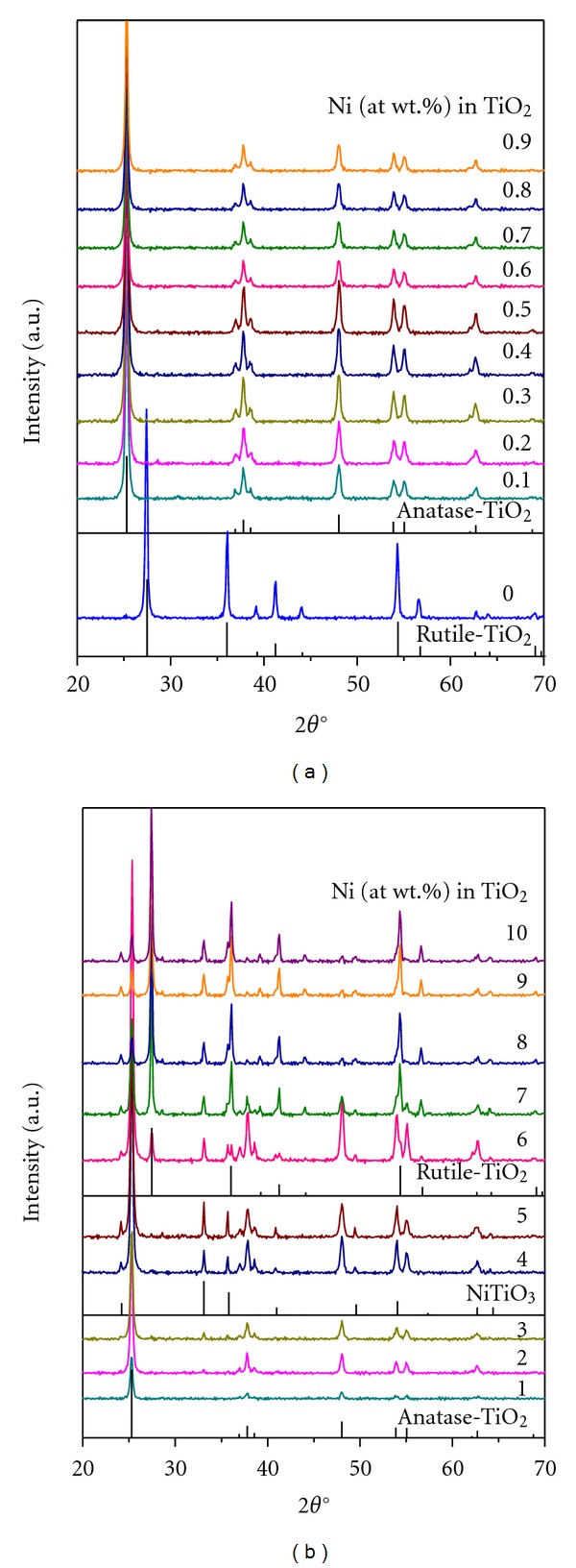
XRD patterns of Ni-doped TiO_2_ powders, where Ni = 0, 0.1 to 0.9 at. wt.% (a), and Ni = 1 to 10 at. wt.% (b), formed at 550°C for 6 h. Rutile TiO_2_: ICDD File number: 03-065-1118; anatase TiO_2_: ICDD File number: 03-065-5714; NiTiO_3_: ICDD File number: 04-006-6640.

X-ray diffraction patterns of Ni-doped TiO_2_ (Ni = 0 to 0.9, and 1 to 10%) powders formed at 550°C for 6 h are presented in Figures 1(a) and 1(b), respectively. For the sake of an easy interpretation, the XRD patterns reported in ICDD files for anatase TiO_2_ (ICDD File number: 03-065-5714), rutile TiO_2_ (ICDD File number: 03-065-1118), and NiTiO_3_ compound (ICDD File number: 04-006-6640) are also presented in these figures. It can be seen that, in general, all the powders formed at 550°C for 6 h are well crystalline materials, and the 0.1% Ni-doping caused transformation of TiO_2_ from completely rutile to completely anatase phase. Anatase is the only phase in the powders doped with Ni up to a concentration of 2.0%. Further increase of Ni from 2 to 3% not only caused an increase in the intensity of lines belonging to anatase phase but also the formation of NiTiO_3_ phase with anatase being the major phase. Up to 5% Ni-doping, only anatase and NiTiO_3_ phases are noted with the former being the major one. However, 6% Ni-doping not only caused an increase in the intensity of lines due to anatase TiO_2_ and NiTiO_3_ phases but also transformation of some portion of TiO_2_ back to rutile phase. There is a gradual increase and decrease in the intensities of rutile and anatase phases, respectively, with the increase of Ni-doping concentration from 6 to 10% in TiO_2_ along with little change in the intensity of lines of NiTiO_3_. The major portion of TiO_2_ powder doped with 10% Ni is rutile and the rest being anatase and NiTiO_3_ phases with almost equal percentages. The major portions of powders doped Ni up to 6% are anatase phase, whereas the major portions of the powders doped with >6% are rutile.

**Figure 2:**
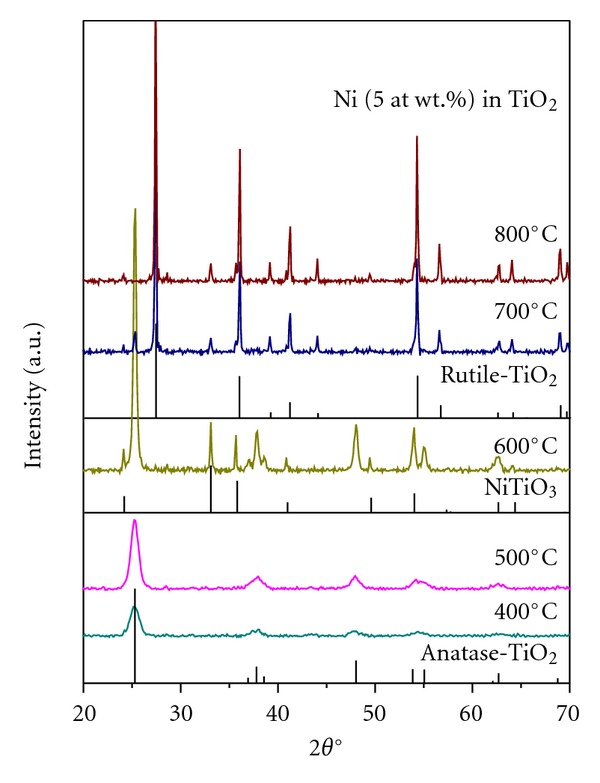
XRD patterns of 5 at. wt.% Ni-doped TiO_2_ powder formed at 400 to 800°C for 6 h. Rutile TiO_2_: ICDD File number: 03-065-1118; anatase TiO_2_: ICDD File number: 03-065-5714; NiTiO_3_: ICDD File number: 04-006-6640.

Figure 2 presents the XRD patterns of 5% Ni-doped TiO_2_ powder calcined at different temperatures ranging from 400 to 800°C for 6 h. The powders formed at 400 and 500°C are poor crystalline materials with only anatase phase, whereas the one formed at 600°C is a well-crystalline anatase material. The powder formed at 600°C also contains some small amounts of NiTiO_3_. However, the powders formed at ≥700°C are almost rutile powders with very minor contents of NiTiO_3_. The reason for stabilizing TiO_2_ in anatase phase upon doping with Ni at lower levels has been attributed to the almost similar ionic radius of Ni^+2^ (0.72 Å) to that of Ti^+4^ (0.68 Å), which was found to replace some portion of Ti^+4^ ions in TiO_2_ lattice [16, 21, 23–30]. The present study suggests that Ni up to 6% stabilizes TiO_2_ in anatase phase and beyond that it transforms TiO_2_ back to rutile phase. This study further suggests that Ni up to a concentration of 2% could replace some portion of Ti^+4^ ions in TiO_2_ lattice or occupy interstitial positions of TiO_2_ crystal structure or stay on the surface of TiO_2_ as a single lamella of unimolecular oxide amorphous monolayer hence no XRD lines belonging to NiO or NiTiO_3_.

The values of BET surface area, crystallite size, and lattice parameters of Ni-doped TiO_2_ (Ni = 0, 0.1, 0.5, 1, 5, and 10%) powders formed at 550°C for 6 h are presented in Table 1. The undoped rutile TiO_2_ powder possesses crystallite size of 17.82 nm and surface area of 23.25 m^2^/g. Upon doping with 0.1% Ni, the crystallite size was decreased to 16.03 nm and the surface area value increased to 40.71 m^2^/g. When dopant concentration was increased to 0.5%, the crystallite size value further decreased to 13.84 nm and the surface area value further increased to 42.70 m^2^/g. These results suggest that Ni below 0.5% doping concentration effectively inhibits TiO_2_ grain growth probably by staying at grain boundaries there by decreasing the crystallite size and increasing the surface area [16, 21, 23–30]. The decrease in grain growth can also be attributed to the formation of Ni–O–Ti bonds in the doped powders, which inhibits the growth of the crystals. However, as the Ni concentration increased further to 1, 5, and 10%, the crystallite size and surface area values increased and decreased, respectively, to 34.57, 32.61, and 13.45 m^2^/g and 17.30, 17.47, and 18.47 nm. These opposite changes could be attributed to the formation of NiTiO_3_ and rutile phases in these latter powders. The “*a*” (= “*b*”) and “*c*” lattice constant values estimated for undoped TiO_2_ are 4.585 Å and 2.964 Å, respectively, whereas, the anatase TiO_2_ powders doped with 0.1, 0.5, 1, and 5% Ni revealed constant “*a*” values of 3.785 Å and varied “*c*” values from 9.452 to 9.715 Å. In a study, Devi et al. [24] reported a gradual elongation of *c*-axis with the increase in the Ni^2+^ concentration from 0.01% to 0.1% in anatase TiO_2_. This confirms the possibility of incorporation of Ni^2+^ ion in place of Ti^4+^ lattice sites. Since the change is only in *c*-axis taking place, while “*a*” (= “*b*”) remains almost constant for the entire range of dopant concentration, it is speculated that Ni^2+^ substitutes for Ti^4+^ preferentially on the body-centered and face-centered lattice sites in tetragonal anatase structure (Table 1). On the other hand, the 10% Ni doped rutile TiO_2_ powder exhibited the values of 4.595 Å for “*a*” (= “*b*”) and 2.958 Å for “*c*”. These values indicate that in comparison to the undoped TiO_2_ rutile powder, the 10% Ni-doped TiO_2_ rutile powder possessed a larger “*a*” lattice parameter value and a lower “*c*” lattice parameter value. These results suggest that in comparison to anatase TiO_2_ crystals, the rutile TiO_2_ crystals accommodate Ni^2+^ ions at different sites. Nevertheless, these estimated lattice parameter values are well comparable with those reported for rutile (ICDD File number: 03-065-1118) and anatase (ICDD File number: 03-065-5714) titania in ICDD Files.

**Figure 3:**
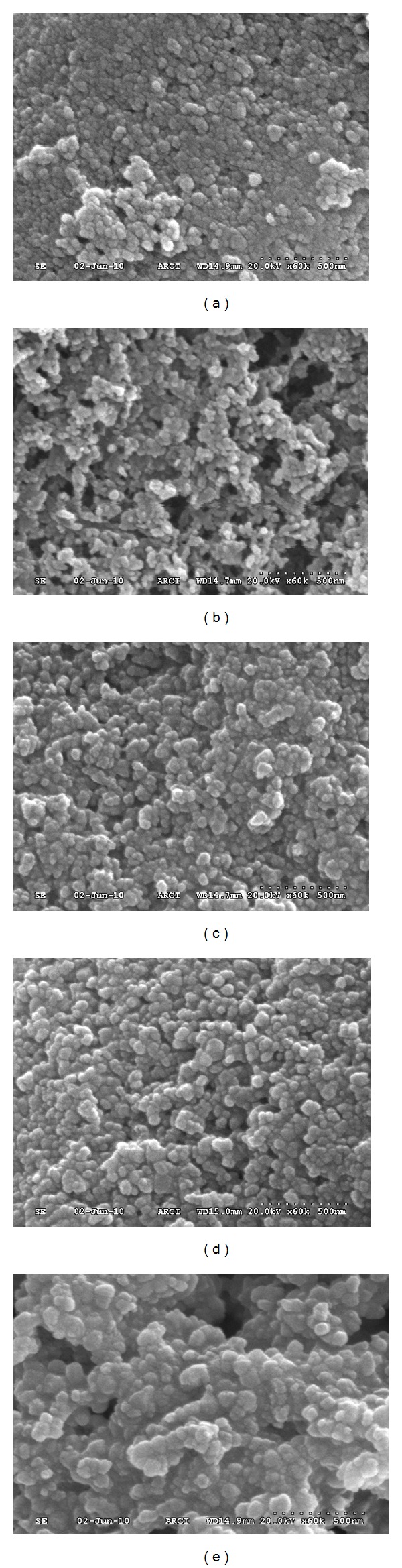
SEM micrographs of Ni-doped TiO_2_ (Ni = 0.1 (a); 0.5 (b); 1 (c); 5 (d); 10 (e) at. wt.%) powders formed at 550°C for 6 h.

**Figure 4:**
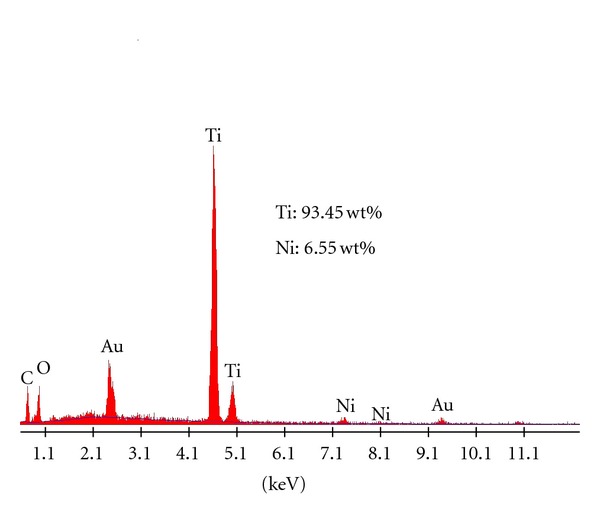
EDAX spectra of 5 at. wt.% Ni-doped TiO_2_ powder formed at 550°C for 6 h.

The SEM micrographs of Ni-doped TiO_2_ (Ni = 0.1, 0.5, 1, 5, and 10%) powders formed at 550°C for 6 h are presented in Figures 3(a)–3(e), respectively. In general, all these powders consisted agglomerates of nanosized spherical primary particles. The powder particles of 0.1 and 0.5% Ni-doped TiO_2_ powders are finer (~50 nm) than those of powders doped with 1, 5, and 10%, which have particles in the range of 100 to 200 nm. The powder doped with 10% Ni consist some portion of particles of about 300 nm. This SEM study suggests that the primary particles sizes could be responsible for the changes seen in the values of BET surface areas of different amounts of Ni-doped TiO_2_ powders. A close look at these micrographs reveal that the degree of agglomeration of TiO_2_ powder particles decreased with the increase of Ni content up to 0.5%, and above that there is an opposite trend in the degree of powders agglomeration. To confirm the chemical composition of the synthesized powders, the TiO_2_ powder doped with 5% Ni was examined by EDAX analysis (Figure 4). This EDAX spectrum confirms that the targeted chemical composition could achieve in the powders.

**Figure 5:**
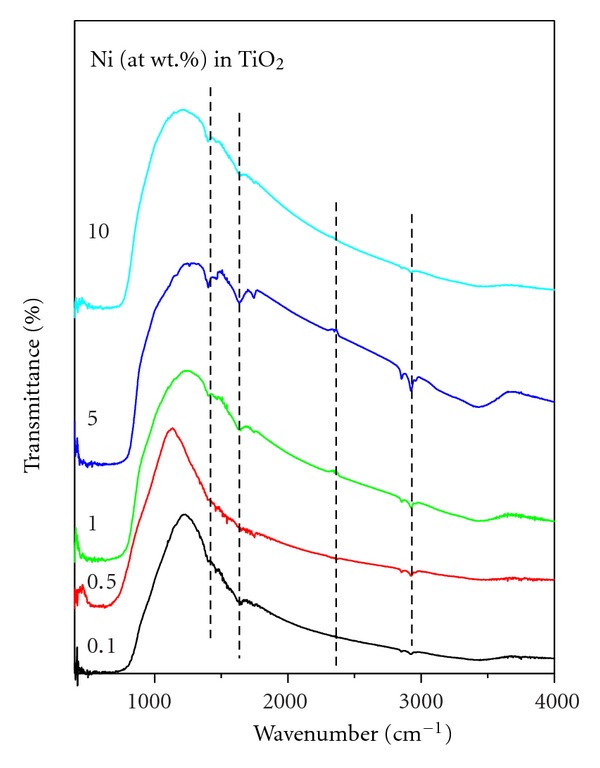
FT-IR spectra of Ni-doped TiO_2_ (Ni = 0, 0.1, 0.5, 1, 5, and 10 at. wt.%) powders formed at 550°C for 6 h.

The FT-IR spectra of Ni-doped TiO_2_ (Ni = 0, 0.1, 0.5, 1, 5, and 10%) powders calcined for 6 h at 550°C are presented in Figure 5. The presence of some weak transmittance bands between 3400 and 3600 cm^−1^ and at 1625 cm^−1^ are seen, which are gradually decreased with the increase of Ni concentration in TiO_2_. These bands are attributed to the stretching vibrations of the O-H groups and the bending vibrations of the adsorbed water molecules, respectively [16, 21, 23–30]. A band between 650 and 830 cm^−1^ is seen which is attributed to different vibrational modes of TiO_2_. Anatase and rutile phases of TiO_2_ exhibit strong FT-IR absorption bands in the regions of 850–650 and 800–650 cm^−1^, respectively [34, 35]. The broad intense band seen below 1200 cm^−1^ is due to Ti–O–Ti vibrations [14]. The shift to the lower wavenumbers and sharpening of the Ti–O–Ti band with an increase of Ni concentration could be attributed to the increase of powders particles sizes [14]. In line to XRD observations, the 5% Ni-doped TiO_2_ powder exhibited additional transmittance bands in comparison to other Ni-doped TiO_2_ powders indicating that the former powder consists of NiTiO_3_ phase. These results match very well with those reported in the literature for Ni-doped TiO_2_ powders and for anatase and rutile phases of TiO_2_ [16, 21, 23–30, 34, 35].

**Figure 6:**
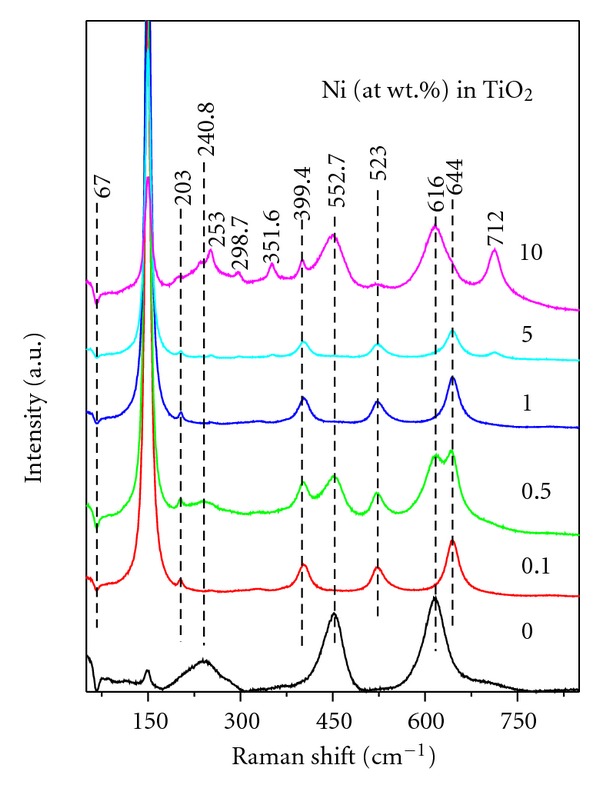
FT-Raman spectra of Ni-doped TiO_2_ (Ni = 0, 0.1, 0.5, 1, 5, and 10 at. wt.%) powders formed at 550°C for 6 h.

Figure 6 shows the Raman scattering spectra of Ni-doped TiO_2_ (Ni = 0, 0.1, 0.5, 1, 5, and 10%) powders calcined for 6 h at 550°C. The tetragonal rutile structure possesses two TiO_2_ molecules in the unit cell with the space group D_4*h*_^14^(*P*4_2_/*mnm*) and the lattice constants *a* = 0.4594 and *c* = 0.2958 nm [45]. There are six atoms in the unit cell, implying a total of 15 ( = 3*N* − 3) vibrational modes. From a group theoretical analysis, it can be shown that these 15 modes have the following irreducible representations: A_1g_ + A_2g_ + A_2u_ + B_1g_ + B_2g_ + 2B_1u_ + E_g_ + 3E_u_. Further, group theory reveals that four modes, A_1g_ + B_1g_ + B_2g_ + E_g_, are Raman active and four modes, A_2u_ + 3E_u_, are infrared active. The other three modes, A_2g_ + 2B_1u_, are neither Raman active nor infrared active. The frequencies of the Raman bands noted for pure undoped TiO_2_ powder (i.e., for rutile) are at 141–157, 181–290, 415–479, 585–650 cm^−1^ (Figure 6). Balachandran and Eror [45] reported six frequencies at 144, 235, 320–360, 448, 612, and 827 cm^−1^ for rutile titania. On the other hand, the conventional crystallographic unit cell of anatase TiO_2_ is body-centred tetragonal (space group D_4*h*_^19^, *I*4_1_/*amd*, with an elongated cell having *a* = 0.3783 and *c* = 0.951 nm) and contains two primitive unit cells, each of which contains two formula units of TiO_2_ [45]. According to the factor group analysis, six modes of anatase TiO_2_, A_1g_ + 2B_1g_ + 3E_g_, are Raman active and three modes, A_2u_ + 2E_u_, are infrared active. One vibration, B_2u_, will be inactive in both infrared and Raman spectra. All of these modes account for the 15 normal modes of vibration. Thus, group theory predicts six Raman active modes for the tetragonal anatase phase: three E_g_ modes centred around 145, 197, and 639 cm^−1^; two B_1g_ modes at 399 and 519 cm^−1^; one A_1g_ mode at 513 cm^−1^ [46]. The frequencies of the Raman bands noted for anatase TiO_2_ (in the powders doped with Ni = 0.1, 0.5, 1, and 5%) are at 108–188, 205, 387–425, 512–543, 625–670 cm^−1^ (Figure 6). In the literature, Raman bands reported for anatase TiO_2_ are at 146, 198, 320, 398, 448, 515, 640, and 796 cm^−1^ [45]. As mentioned above, the band at about 146 cm^−1^, the strongest of all the observed bands and the bands at 198, and 640 cm^−1^ are assigned to the E_g_ modes and the band at 398 cm^−1^ to the B_1g_ mode. The doublet band at 515 cm^−1^ is assigned to A_1g_ and B_1g_ modes [45]. Thus, these results are well comparable with those reported for anatase and rutile phases of TiO_2_ in the literature [47]. The additional frequencies of the Raman bands observed for 10% Ni-doped TiO_2_ powder at room temperature belonging to NiTiO_3_ are at about 253, 298.7, 351.6, 552.7, and 712 cm^−1^. For ilmenite-type of compounds such as NiTiO_3_, 10 Raman active modes (5A_g_ + 5E_g_) were observed and assigned to *C*_3*i*_^2^ symmetry and $R\bar{3}$ space group [30, 35]. In a study, Chuang et al. [29] observed peaks at 226, 246, 289, 343, 392, 464, 609, and 704 cm^−1^ for NiTiO_3_ powder formed at ~550°C for 2 h. The some of the missing peaks of NiTiO_3_ in the present study can be attributed to their lower intensities. These results confirm that the anatase structure is retained even after doped with 5% Ni in TiO_2_. Thus, these results are in good agreement with those revealed by XRD and FTIR studies. The absence of characteristic vibrational modes of NiO in the Raman spectrum suggests that the formed nickel oxide could react with TiO_2_ leading to the formation of NiTiO_3_ [16, 21, 23–30].

**Figure 7:**
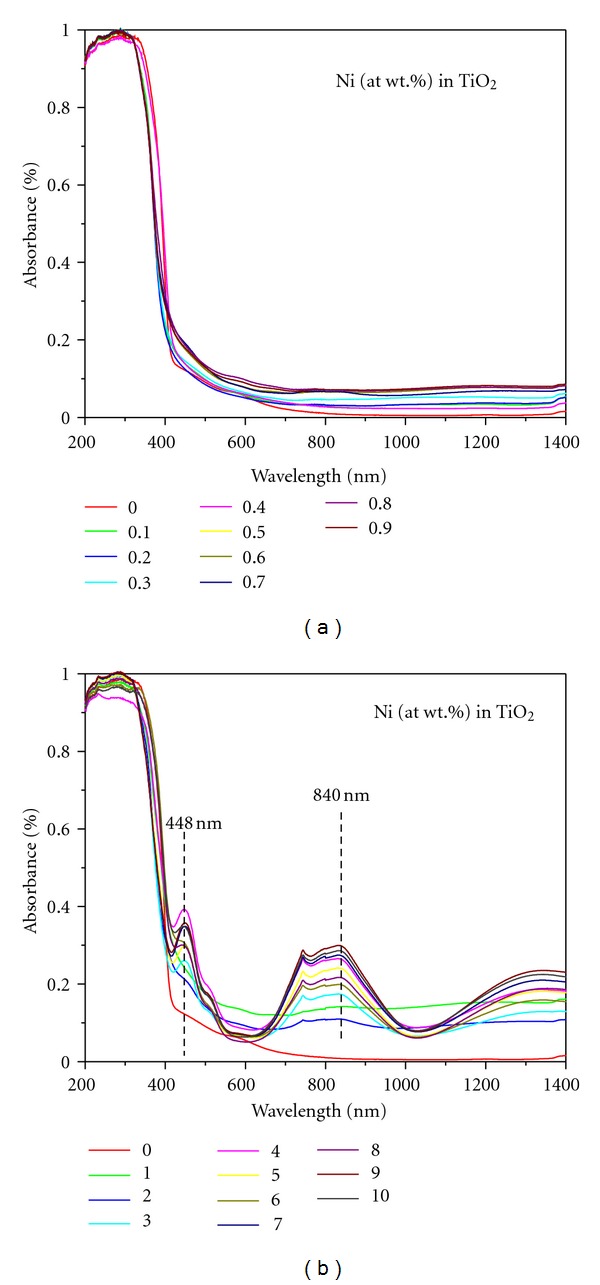
UV-Vis diffuse reflectance spectra (DRS) of Ni-doped TiO_2_ (Ni = 0, 0.1 to 0.9 (a) and = 0, 1 to 10 at. wt.% (b)) powders formed at 550°C for 6 h.

The UV-Vis diffuse reflectance spectra of Ni-doped TiO_2_ (Ni = 0, 0.1, 0.2, 0.3, 0.4, 0.5, 0.6, 0.7, 0.8, and 0.9, and 1, 2, 3, 4, 5, 6, 7, 8, 9, and 10%) powders calcined at 550°C for 6 h are presented in Figures 7(a) and 7(b), respectively. It can be seen that pure rutile (the undoped) and anatase (doped with 0.1% Ni) TiO_2_ powders (Figure 7(a)) show absorption spectra consisting a single broad intense absorption at around 400 nm (i.e., in the UV range) occurred due to the charge transfer (CT) from the valence band (mainly formed by 2p orbitals of the oxide anions) to the conduction band (mainly formed by 3d t_2g_ orbitals of the Ti^4+^ cations) [48]. The 0.1% Ni-doped TiO_2_ powder exhibited the spectra just like the one exhibited by P-25 TiO_2_ (Degussa) powder [48]. The DRS spectra of Ni-doped TiO_2_ (Ni = 0.2 to 5%) powders reveal two wide absorption bands in the wavelength ranges of 400–550 nm and 650–1000 nm (visible region) with two *λ*_max⁡_ peaks (a narrow one at about 448 nm and a broad one 840 nm). The sharpness and the degree of absorbance of these latter peaks increased with the increase of Ni-doping concentration in TiO_2_. The sharpness of peaks has started increasing after doping with >3% Ni (Figure 7(b)). In a study, two apparent absorption peaks of NiTiO_3_ at around 450 and 515 nm formed due to the crystal field splitting of 3d^8^ orbital and due to charge transfer from Ni^2+^→Ti^4+^, respectively, are reported [16]. The observed peaks could be identified at the onset of 490 and 560 nm corresponding to the subbandgaps of 2.53 and 2.21 eV. Another broad absorption edge situated at about 410 nm (3.02 eV) was also noted in their study and it was assigned to the optical bandgap charge transfer transition from O^2−^→Ti^4+^. Since, NiTiO_3_ samples are heavily coloured, in the present study, the reflection spectra were dominated by the broad intense absorption in the visible region at around 740 nm. The doping of various transitional metal ions into TiO_2_ could shift its optical absorption edge from UV into visible-light range (i.e., red shift) [49]. Thus, these results are in good agreement with those reported for NiTiO_3_− and Ni-doped TiO_2_ powders in the literature [16, 21, 23–30].

**Figure 8:**
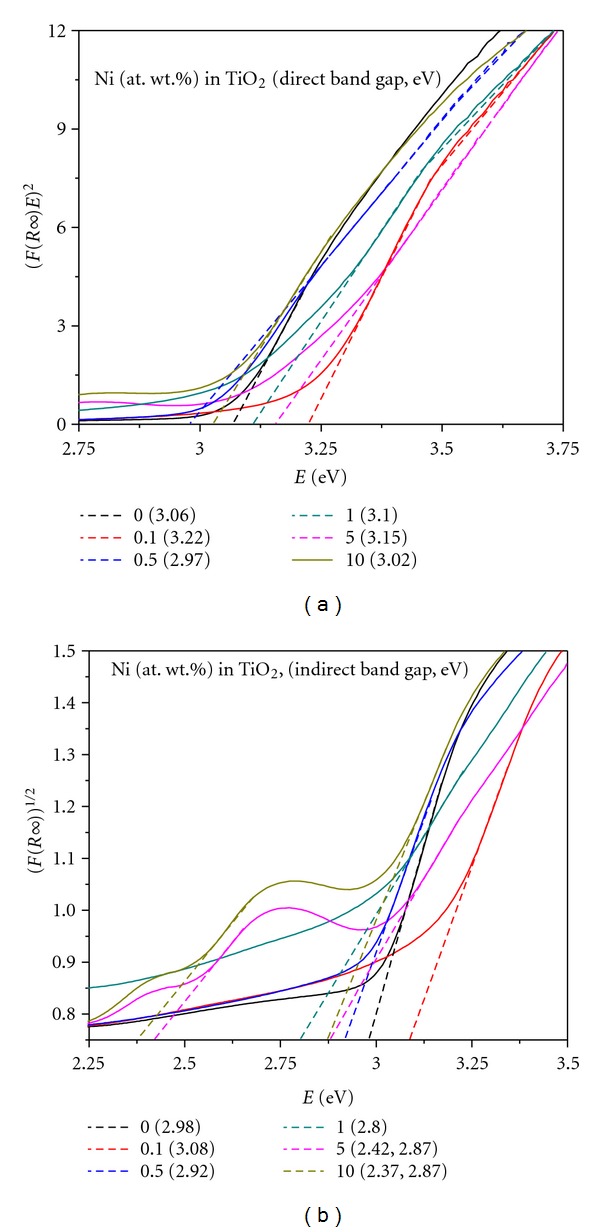
Transformed diffuse reflectance spectra of Ni-doped TiO_2_ (Ni = 0, 0.1, 0.5, 1, 5, and 10 at. wt.%) powders formed at 550°C for 6 h showing direct bandgap (a) and indirect bandgap (b) energy values.

In order to establish the type of band-to-band transition in Ni-doped TiO_2_ (Ni = 0.1, 0.5, 1, 5, and 10%) powders formed at 550°C for 6 h, the absorbance data of DRS spectra was fitted into equations for both indirect and direct bandgap transitions [32, 33].  Figure 8(a) shows the *(F(R∞)E)^2^* versus *E*_phot_ plot for direct transition and Figure 8(b) shows the *(F(R∞)E)^1/2^* versus *E*_phot_ plot for indirect transition. The Kubelka-Munk function *F*(*R∞*) is equivalent to absorbance in these DRS spectra and *E*_phot_ is the photon energy, *E*_phot_ = (1239/*λ*) eV, where *λ* is the wavelength in nm [40]. The value of *E*_phot_ extrapolated to *F(R∞)E *= 0, which gives an absorption energy, corresponds to a bandgap *E*_*g*_ [32, 33]. A close look at these graphs reveals that all the powders possess indirect bandgap transitions. The calculated *E*_*g*_ values of these powders are presented in Table 2. It can be seen that as the Ni-doping concentration increased, the TiO_2_ bandgap energy is gradually decreased, which is in consistent with the red shift of absorption edge observed in DRS spectra (Figure 8). These red shifts can be attributed to the charge transfer transitions between the metal ion *d* electrons and the conduction or valence band of TiO_2_. Based on *E*_*g*_ and absorption edge values obtained, the pure TiO_2_ is expected to be active under UV irradiation, and the Ni-doped TiO_2_ (Ni = 0.1, 0.5, 1, 5, and 10%) powders under visible light [50].

**Figure 9:**
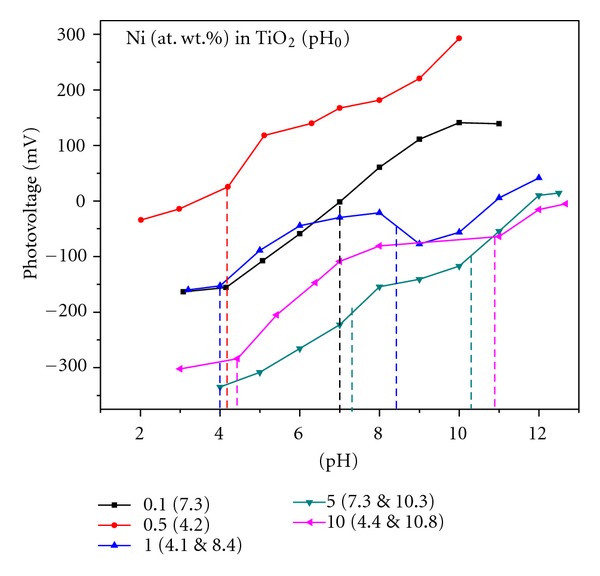
Dependence of photovoltage (V versus Ag/AgCl) of Ni-doped TiO_2_ (Ni = 0.1, 0.5, 1, 5, and 10 at. wt.%) powders formed at 550°C for 6 h on pH of electrolyte.

**Figure 10:**
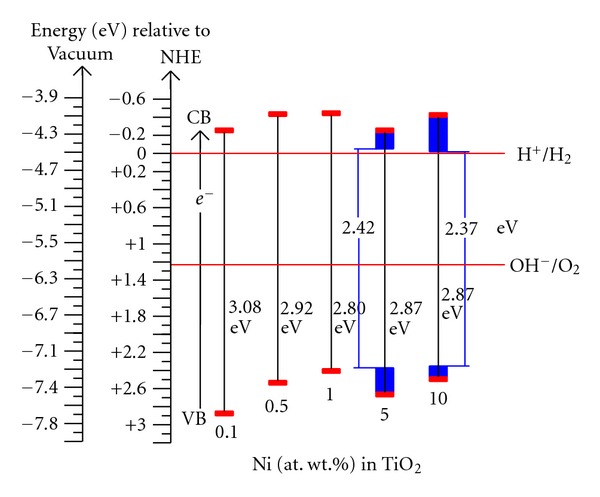
The electrochemical potentials (versus NHE) of band edges of Ni-doped TiO_2_ (Ni = 0.1, 0.5, 1, 5 and 10 at. wt.%) powders (formed at 550°C for 6 h) at pH 7.

In order to find out whether a shift of the valence or conduction band edge is responsible for the decrease of the bandgap energy of TiO_2_ with the increase of Ni-doping concentration in it, the position of the quasi-Fermi level of electrons (_*n*_ *E*_F_*) (i.e., the flat-band potential values, *U*_fb_) was determined by measuring the photovoltages of Ni-doped TiO_2_ powders as a function of the suspension pH value (Figure 9). From the pH value of the inflection point (pH_0_), the quasi-Fermi level at pH 7 could be calculated (Table 2) [32, 33]. An increase in Ni-dopant concentration caused no apparent trend in the position of _*n*_ *E*_F_*. This unusual trend could be attributed to the changes took place in the XRD phases of these powders with the increase of Ni-dopant concentration in TiO_2_. A value of −0.52 V for TiO_2_ at pH 7 measured by slurry method [33], −0.58 V for an anatase single crystal measured by Mott-Schottky method [51] and −0.47 V for anatase powder also measured by slurry method [52] were reported in the literature. The values measured in the present study are slightly different from those reported values [32, 33]. These differences could be attributed to dopant Ni in TiO_2_ and to the differences in their methods of preparation. Assuming that the distance between the quasi-Fermi level of electrons and the conduction band edge is decreasing with the increase of Ni in TiO_2_, one can locate the position of the valence band edge by adding the bandgap energy to the quasi-Fermi level value [32, 33]. Potential values of 2.85, 2.51, 2.38, 2.64, and 2.47 V (versus NHE) for TiO_2_ powders doped with Ni = 0.1, 0.5, 1, 5, and 10%, respectively, could be calculated at pH 7 (Figure 10). It can also be seen that the NiTiO_3_, an additional phase present in both 5 and 10% Ni-doped TiO_2_ powders, is caused for the development of separate energy states with bandgap energies of 2.42 and 2.37 eV, respectively (Figure 10(a)). This information is further supported by separate pH values of the inflection points (pH_0_) measured for these latter two powders. These separate energy states could cause the further reduction in the bandgap energies of TiO_2_ powders to 2.42 and 2.37 eV, respectively (Figure 10). These bandgap energies are corresponding to wavelengths belonging to visible light of major portion of the sun light reaching the surface of the earth. The reduction and oxidation potentials of water (i.e., the light reaction of natural photosynthesis) lie at 0 and 1.23 eV (versus NHE), respectively, as shown with horizontal dashed lines in Figure 10. Thus, the measured flat band potentials and bandgap energy values suggest that these synthesized Ni-doped TiO_2_ powders could be the candidate members of choice for artificial photosynthesis reactions.

Table 2 also lists the measured values of zeta-potentials (*ζ*) of Ni-doped TiO_2_ (Ni = 0.1, 0.5, 1, 5 and 10%) powders formed at 550°C for 6 h. It can be seen that there is a gradual increase in the zeta-potential values of TiO_2_ from −21.1 ± 2.22 mV to −28.8 ± 2.40 mV with the increase of Ni-dopant concentration from 0.1 to 10%. The isoelectric point of TiO_2_ lies in pH less than 6.8 [51, 52]. The solution at pH 7 renders TiO_2_ powder surface a negative charge, resulting in negative *ζ* values. The change of *ζ* values with increase of Ni-dopant concentration suggests that the isoelectric point of this powder is changing with the increase of Ni concentration. Development of charge on TiO_2_ powder surface in aqueous medium can be shown with the following equations.

(2) $$\begin{array}{c} \text{Ti-OH}+{\text{H}}^{+}⟷\text{Ti-}{{\text{OH}}_{2}}^{+},\\ \text{Ti-OH}+\text{-O}{\text{H}}^{-}⟷\text{Ti-}{\text{O}}^{-}+{\text{H}}_{2}\text{O}. \end{array}$$

It is generally accepted that the surface of the TiO_2_ powder exists in the form of TiOH groups. These hydroxyl groups dissociate into water and confer to the particles a surface charge as shown in (2). These equations further suggest that the solution pH also possesses a strong influence on the surface catalytic reactions of TiO_2_ powders in aqueous medium. Since these powders possess negative zeta-potentials, it is expected that the reactions of organic molecules/reactants having positive charge would be higher on the surfaces of Ni-doped TiO_2_ powders [39, 40].

**Figure 11:**
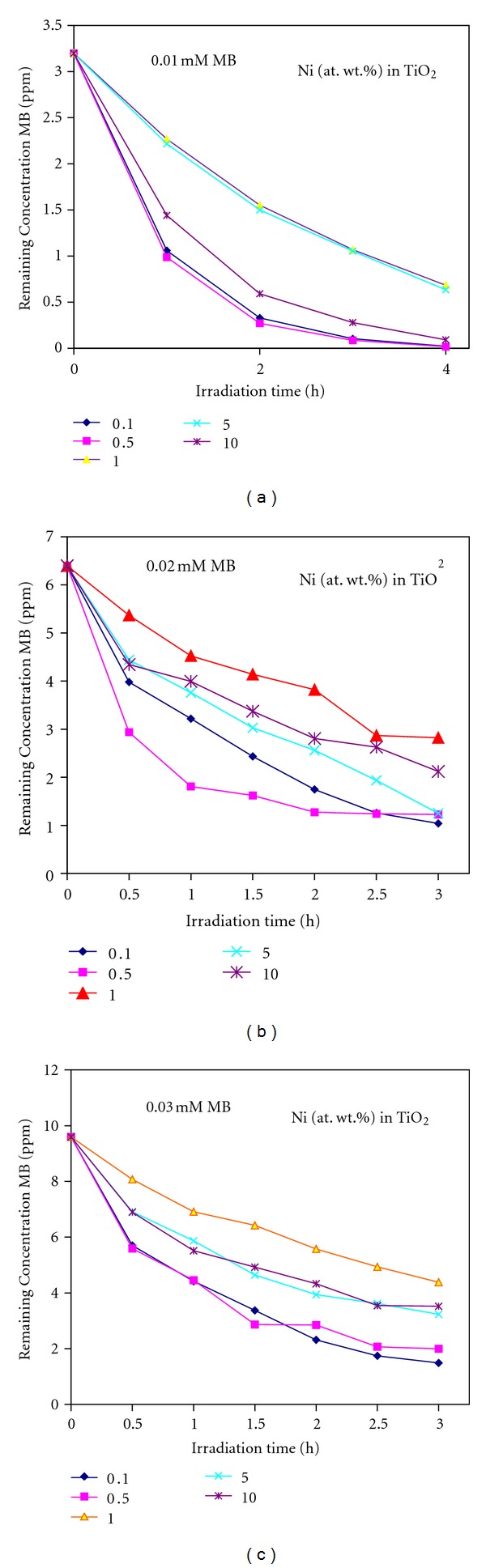
Effect of the concentration of methylene blue (MB) on photocatalytic ability of Ni-doped TiO_2_ (Ni = 0.1, 0.5, 1, 5, and 10 at. wt.%) powders formed at 550°C for 6 h. (a) 0.01 mM, (b) 0.02 mM, and (c) 0.03 mM MB solutions.

**Figure 12:**
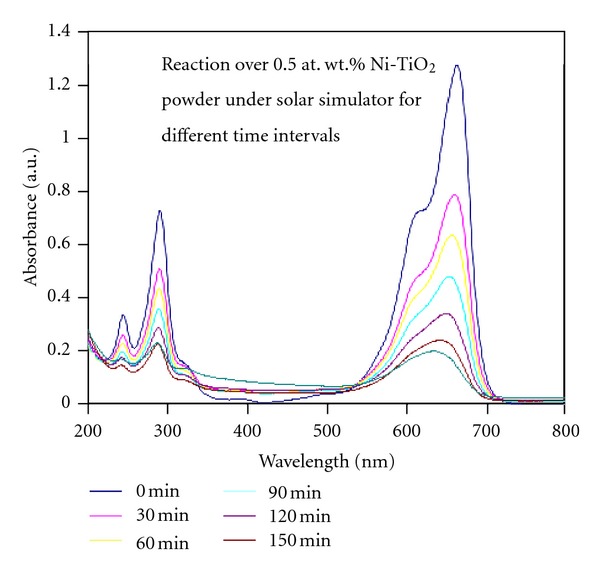
Photocatalytic degradation of methylene blue (MB) from 0.02 mM concentrated aqueous solution over 0.5 at. wt.% Ni-doped TiO_2_ powder formed at 550°C for 6 h under simulated solar light having a power density of about 44 mW/cm^2^ for different time intervals.

The methylene blue (MB) degradation experiments in water under simulated solar light were conducted to assess the photocatalytic efficiency of Ni-doped TiO_2_ (Ni = 0.1, 0.5, 1, 5 and 10%) powders. Figures 11(a), 11(b), and 11(c) shows the remaining concentrations of 0.01, 0.02, and 0.03 mM aqueous MB solutions, respectively, as a function of reaction time. It can be seen that among the five powders tested, only 0.5% Ni-doped TiO_2_ powder showed the highest photocatalytic activity and as the concentration of Ni further increased in TiO_2_, the photocatalytic efficiency is gradually decreased. Furthermore, the photocatalytic efficiency of these catalysts also decreased with the increase of MB concentration in the aqueous solution. The degradation behavior of 0.02 mM MB solution over 0.5% Ni-doped TiO_2_ powder with reaction time could be seen from Figure 12. In order to assess the rate of photocatalytic degradation of MB over Ni-doped TiO_2_ (Ni = 0.1, 0.5, 1, 5, and 10%) powders, the observed MB degradation results were kinetically analyzed by following Langmuir-Hinshelwood rate constant equations [19]. Langmuir-Hinshelwood (3) described the relationship between the initial degradation rate (*r*) and the initial concentration (*C*) of the organic substrate for heterogeneous photocatalytic degradation [19]. The model can be written as follows:

(3) $$\begin{array}{c} r=\frac{-dC}{dt}=\frac{kK_{\text{ads}}C}{1+K_{\text{ads}}C}=k_{\text{obs}}C, \end{array}$$

where

(4) $$\begin{array}{c} k_{\text{obs}}=\frac{kK_{\text{ads}}}{1+K_{\text{ads}}C} \end{array},$$

(5) $$\begin{array}{c} \frac{1}{k_{\text{obs}}}=\frac{1}{kK_{\text{ads}}}+\frac{C}{k} \end{array},$$

(6) $$\begin{array}{c} -\ln\frac{C_{t}}{C_{0}}=k_{\text{obs}}t. \end{array}$$

*C*_0_ (ppm) is the initial concentration of MB, *C*_*t*_ (ppm) is the remaining concentration after *t* (h) time irradiation, *K*_ads_ is the Langmuir-Hinshelwood adsorption equilibrium constant (ppm^−1^), and *k* is the pseudofirst-order rate constant relating to TiO_2_ surface reaction (ppm h^−1^).

**Figure 13:**
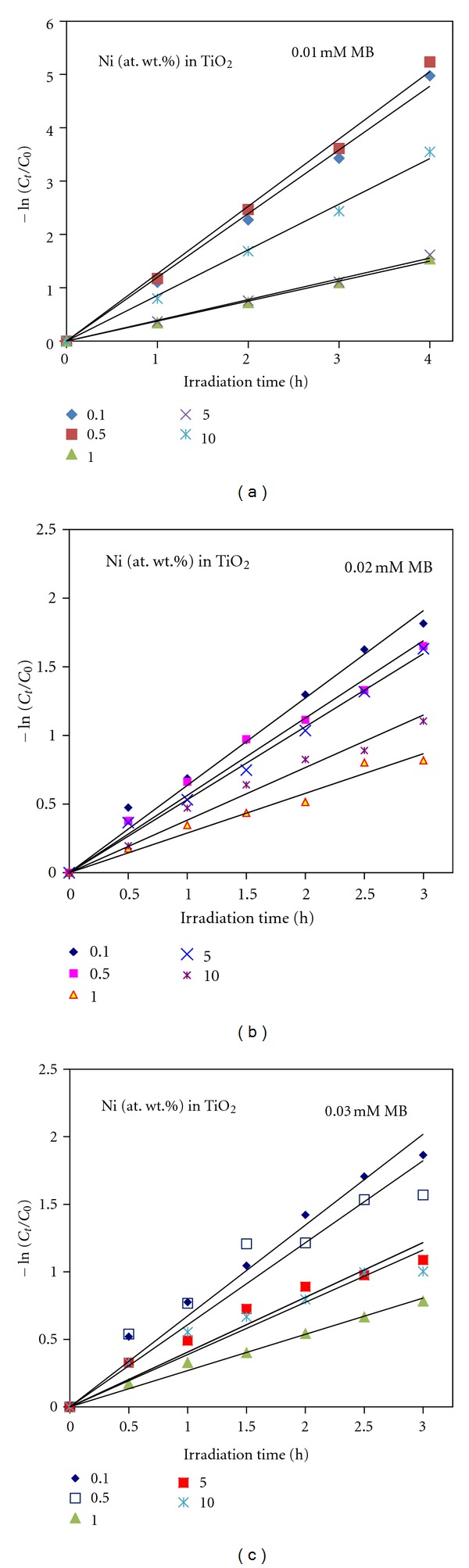
Linear transforms: −In⁡(*C*_*t*_/*C*_0_) versus irradiation time for methylene blue (MB) under simulated solar light over Ni-doped TiO_2_ (Ni = 0.1, 0.5, 1, 5, and 10 at. wt.%) powders formed at 550°C for 6 h. (a) 0.01 mM, (b) 0.02 mM, and (c) 0.03 mM MB solutions.

The *k* observed (*k*_obs)_ values for each initial concentration were estimated from the slopes of straight line obtained by plotting −ln⁡⁡(*C*_*t*_/*C*_0_) versus irradiation time as shown in Figure 13 and in Table 3. When initial concentrations were plotted versus 1/*k*_obs_, the rate constant (*k*) and the adsorption equilibrium constant *K*_ads_ were calculated. Equation (5) can be identified as *Y* = *mX* + *C*, where, *C* = 1/*kK*_abs_, *m* = 1/*k*, *Y* = 1/*k*_obs_, and *X* is the initial concentration (*C*_0_) of MB. Using the Equation (3) and the data of Table 3, the initial degradation rates (*r*) of MB over Ni-doped TiO_2_ powders were calculated. It can be clearly seen that the Langmuir-Hinshelwood adsorption equilibrium constant and the initial degradation rate of MB over these powders decrease with the increase of Ni concentration in TiO_2_ as well as with the increase of MB concentration in the reaction mixture. It is interesting to see that the 10% Ni-doped TiO_2_ powder shows the highest Langmuir-Hinshelwood adsorption equilibrium constant despite its lower degradation rate values. However from the data of Figures 11 and 13, it is clear that the TiO_2_ powder doped with 0.5% Ni exhibits the highest photocatalytic activity for MB degradation under simulated solar light.

**Figure 14:**
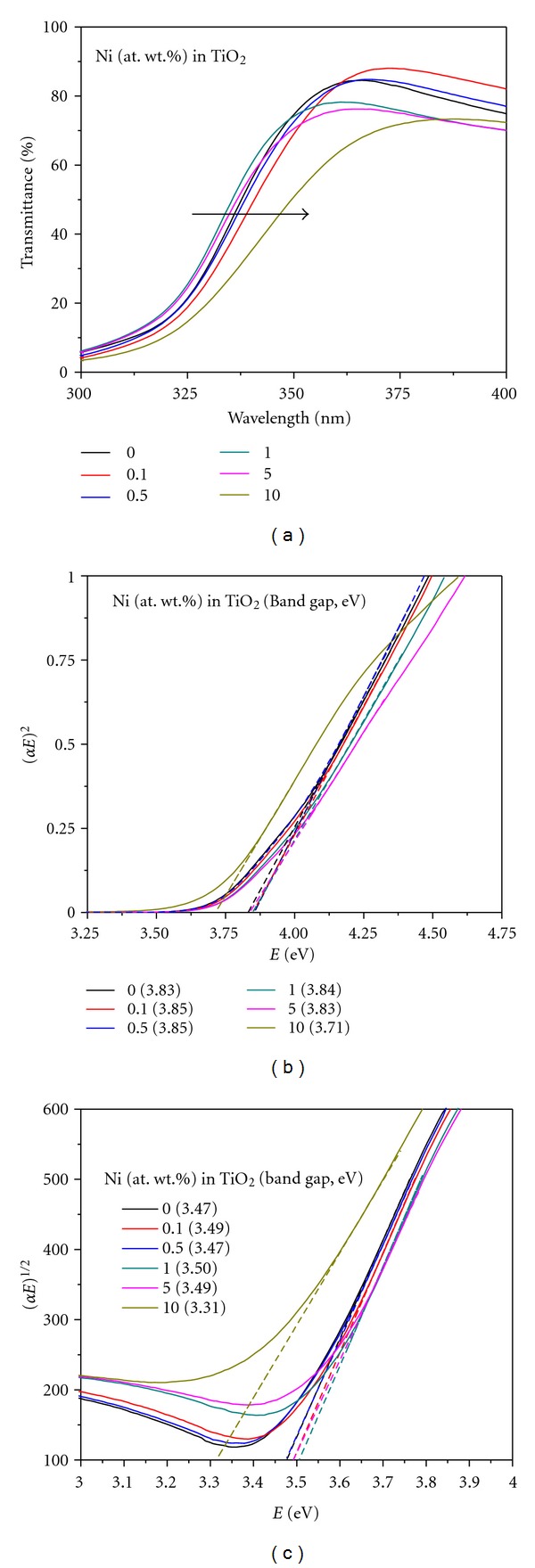
(a) UV-vis transmittance spectra of Ni-doped TiO_2_ (Ni = 0, 0.1, 0.5, 1, 5, and 10 at. wt.%) thin films formed 600°C for 30 min, and their corresponding transformed spectra showing direct bandgap (b) and indirect bandgap (c) values.

Figure 14 shows typical UV-Vis transmittance spectra recorded on optically transparent Ni-doped TiO_2_ thin films deposited on fused silica (FS) glass substrates between 200 and 800 nm wavelength. It can be seen that the pure and Ni (= 0.1, 0.5, 1, and 5%) doped TiO_2_ films are >80% transparent in the visible region and start absorbing light at >360 nm, whereas the 10% Ni-doped TiO_2_ thin film observes light right from 370 nm. With increasing nickel concentration in TiO_2_, the absorption edge shifts towards higher wavelength side indicating the decrease of the bandgap of the films (Figure 14(a)). It can also be clearly seen that the absorption data of these films fit very well into the equations of direct bandgap transition in comparison to those of indirect bandgap transitions (Figures 14(b) and 14(c)). These results indicate that in line to the powder compositions, the film compositions also possess the indirect bandgap transitions [50]. However, it is interesting to see that the values of bandgap energies of these thin films are higher in comparison to those of their corresponding powder counterparts (Table 2). It has been reported that as the particle size decreases to a certain critical size, its bandgap energy increases [50]. Since the thickness of these thin films has been estimated to be <180 nm (by SEM analysis not shown here) containing much finer particles/grains, the calculated values of bandgap energies have been found to be higher side.

**Figure 15:**
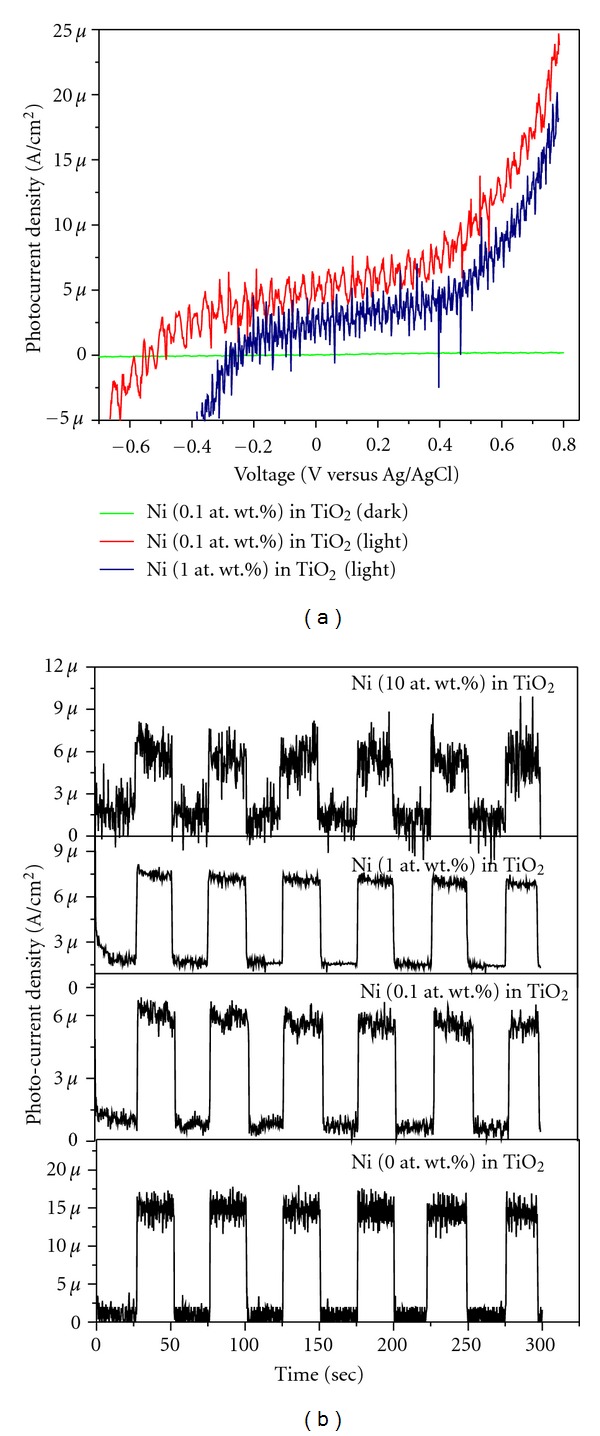
(a) Current-potential curves for photoelectrodes prepared from Ni-doped TiO_2_ (Ni = 0.1 and 1 at. wt.%) thin films formed at 550°C for 30 min on fluorine doped tin oxide (F : SnO_2_, FTO) coated soda lime glass substrates and (b) their corresponding chronoamperometric currents at 0.4 V (versus Ag/AgCl). Electrolyte: 1 M NaOH solution. Light wavelength: 350 nm and power density 1.039 mW/cm^2^.

Figures 15(a) and 15(b) show the typical photocurrents generated by the illumination of Ni-doped TiO_2_ (Ni = 0, 0.1, 0.5, 1, 5, and 10%) thin films deposited on FTO-coated conductive soda-lime glass substrates under a light radiation of 350 nm wavelength and their corresponding typical chronoamperometric currents, respectively. It can be seen that these thin films show appreciable photocurrents indicating the existence of stable PEC active films over conductive FTO substrates. These photocurrents increased with electrode potential, and throughout the measured voltage. It can also be seen that the Ni-doped TiO_2_ thin films show the n-type semiconductivity and the highest photocurrent for 0.1% Ni-doped TiO_2_ thin film. This indicates that the photogenerated electron-hole pairs were separated more efficiently in 0.1% Ni-doped TiO_2_ in comparison to other powders. The incident monochromatic photon to current conversion efficiency (IPCE), defined as the number of electrons generated by light in the circuit divided by the number of incident photons, is evaluated at 350 nm by the means of (7):

(7) $$\begin{array}{cc} & \text{IPCE}\;\left(\text{\%}\right)\\ & \;=1240\times i_{\text{sc}}\left(\text{A}\;{\text{cm}}^{-2}\right)\times 100/\lambda \left(\text{nm}\right)\times I_{\text{inc}}\left(\text{W}\;{\text{cm}}^{-2}\right), \end{array}$$

where *i*_sc_ is the short-circuit photocurrent density, *I*_inc_ is the intensity of the corresponding monochromatic light, and *λ* the irradiation wavelength [2, 3]. The calculated IPCE values for 0.1 and 1% Ni-doped TiO_2_ electrodes were estimated to be 1.690 and 1.225%, respectively. A close look at Figure 15(a) reveals that the flat band potentials for 0.1 and 1% Ni-doped TiO_2_ thin films are about −0.67 and −0.35 V (versus Ag/AgCl) in comparison to −0.23 and −0.41 V (versus NHE) values noted for their corresponding powder compositions measured by slurry method (Table 2 and Figure 10). The slight differences noted in these measured flat band potentials could be attributed to the materials physical properties instead of chemical composition as the former ones are thin films and the other ones are micron-sized powders containing the nanosize primary particles [2]. These results indicate that Ni-doped TiO_2_ exhibits n-type semiconducting behavior. It has been suggested that in order to fully convert the potential visible light absorbing capability of Ni-doped TiO_2_ systems into photocurrents as required by artificial photosynthesis reaction, the TiO_2_ could be codoped with Ni in combination with another suitable element. This is the topic of ongoing research in our laboratory.

## 4. Conclusions

The following conclusions can be drawn from the above study: chemically homogeneous and fully crystalline Ni-doped TiO_2_ (Ni = 0 to 10 at. wt.%) powders and thin films can be prepared following coprecipitation and sol-gel dip coating techniques, respectively. The TiO_2_ powder formed from TiCl_4_ in a coprecipitation route exists in the form of crystal pure rutile phase, whereas the TiO_2_ powders doped with Ni up to 6% exist mainly in anatase phase. Even 0.1% Ni is sufficient to transform TiO_2_ from fully rutile to fully anatase phase and to enhance its BET surface area value from 23.25 to 40.71 m^2^/g. The dopant Ni confers a red shift to light absorbing nature of TiO_2_ and reduces its bandgap energy significantly so that it can absorb energy from a major portion of visible light. The 9 and 10% Ni-doped TiO_2_ powders show maximum absorption of visible light at wavelengths of 448 and 840 nm. Pure as well as Ni-doped TiO_2_ powders and thin films possess indirect bandgap energy transitions. Among various Ni-doped TiO_2_ materials, the one doped with 0.1% Ni shows the highest photocurrent and the one doped with 0.5% Ni shows the highest photocatalytic activity for methylene blue (MB) degradation under simulated solar light. The Ni-doped TiO_2_ powders follow the Langmuir-Hinshelwood first-order rate constant relationship in the photocatalytic MB degradation reactions. The Ni-doped TiO_2_ powders possess flat band potentials suitable for artificial photosynthesis reaction. Though, NiO is a p-type semiconducting material, the Ni-doped TiO_2_ materials exhibit n-type semiconducting behavior.

## Figures and Tables

**Table 1:** BET surface area and crystallite size values of Ni-doped TiO_2_ (Ni = 0, 0.1, 0.5, 1, 5, and 10 at. wt.%) powders formed at 550°C for 6 h.^†^

Ni dopant (at. wt. %)	BET surface area (m^2^/g)	Crystallite size (nm)	Lattice parameter (Å)	
			*a* = *b*	*c*
0.0	23.25	17.82	4.585	2.964 (rutile)
0.1	40.71	16.03	3.785	9.452 (anatase)
0.5	42.70	13.84	3.785	9.457 (anatase)
1.0	34.57	17.30	3.785	9.715 (anatase)
5.0	32.61	17.47	3.785	9.457 (anatase)
10.0	13.45	18.47	4.595	2.958 (rutile)

^†^The values are arrived as detailed in the experimental section.

**Table 2:** Photoelectrochemical and band-gap energy data of Ni-doped TiO_2_ (Ni = 0.1, 0.5, 1, 5, and 10 at. wt.%) powders formed at 550°C for 6 h.^†^

Ni (at. wt.%) in TiO_2_	Zeta potential (mV) at pH~7	pH_0_^[a]^	*E*_bg_ [eV]^[b]^		*U*_fb_ (versus NHE)^[a]^ calculated for pH 7 [V]^[c]^
			Direct	Indirect	
0.1	−21.1 ± 2.22	7.3	3.22	3.08	−0.23
0.5	−21.3 ± 2.68	4.2	2.97	2.92	−0.41
1	−21.8 ± 2.65	4.1	3.10	2.80	−0.42
5	−25.6 ± 2.22	7.3 & 10.3	3.15	2.42 & 2.87	−0.23 & −0.05
10	−28.8 ± 2.40	4.4 & 10.8	3.02	2.37 & 2.87	−0.40 & −0.02

^†^The values are arrived as detailed in the experimental section.

^[a]^Measured according to [32, 33]. ^[b]^These values measured for powders by following DRS spectra, and the reproducibility was better than ±0.01 eV. ^[c]^Reproducibility was better than ±0.01 V.

**Table 3:** The initial concentrations of methylene blue (MB), the initial degradation rate (*r*), the Langmuir-Hinshelwood adsorption equilibrium constant (*K*_ads_), and the pseudo-first-order rate constant (*k*) of methylene blue over Ni-doped TiO_2_ (Ni = 0.1, 0.5, 1, 5, and 10 at. wt.%) powders formed at 550°C for 6 h.^†^

Ni (at. wt.%) in TiO_2_	*C*_0_ of MB (ppm)	*K*_obs_ (h^−1^) (±0.020)	*M* (slope)	*C* (intercept)	*K*_ads_ (× 10^−2^ ppm^−1^)	*k* (ppm h^−1^)
0.1	3.198 (0.01 mM)	1.195	0.101	0.647	15.61	9.90
	6.397 (0.02 mM)	0.636				
	9.595 (0.03 mM)	0.672				
0.5	3.198	1.263	0.133	0.549	24.22	7.52
	6.397	0.563				
	9.595	0.607				
1	3.198	0.389	0.181	2.093	8.64	5.52
	6.397	0.289				
	9.595	0.268				
5	3.198	0.531	0.122	1.556	7.84	8.19
	6.397	0.405				
	9.595	0.375				
10	3.198	0.856	0.221	0.705	31.34	4.52
	6.397	0.383				
	9.595	0.387				

^†^The values are arrived as detailed in the experimental section.
